# Analyzing Goals of Care in Advanced Cancer Patients and Their Family Caregivers: Evidence-based Research

**DOI:** 10.32648/2577-9516/2/1/003

**Published:** 2018-04-18

**Authors:** Sara L. Douglas, Amy R. Lipson, Barbara J. Daly

**Affiliations:** 1 Arline H. & Curtis F. Garvin Professor, FPB School of Nursing, Case Western Reserve University, 10900 Euclid Ave, Cleveland, OH 44106, USA; 2 Senior Research Associate, FPB School of Nursing, Case Western Reserve University, 10900 Euclid Ave, Cleveland, OH 44106, USA; 3 The Gertrude Perkins Oliva Professor in Oncology Nursing, FPB School of Nursing, Case Western Reserve University, 10900 Euclid Ave, Cleveland, OH 44106, USA

**Keywords:** Goals of Care, Advanced Cancer, Caregiver

## Abstract

**Objective:**

The study objective was to determine concordance between patients and family caregivers’ preferences for quality or length of life over time.

**Background:**

Patients with advanced cancer are confronted with difficult decisions throughout their course of treatment and at end of life (EOL). These decisions can be influenced by their family caregivers’ preferences for the patient’s cancer treatment.

**Methods:**

Using a longitudinal, descriptive study design from an on-going study, data were collected on an adult sample of patients with advanced stage GI or lung cancers and their family caregivers (n=237). Using a one item visual analog scale (0–100 with higher number indicating a preference for length of life over quality of life), patients and family caregivers were asked “regarding your/your loved one’s care, what is most important to you right now?” Data were collected every 3 months until 15 months or patient’s death.

**Results:**

At enrollment, the preference scores between patients (48.5) and family caregivers (42.6) were closely aligned. At the last assessment prior to death, these scores diverged with the caregivers favoring goals associated with quality of life over length of life (p=.02).

**Discussion:**

Patients and family caregivers have differing preferences and these goals of care can change over time. Attention to these differences could be used to guide conversations between patients and family caregivers regarding preferences at EOL.

## Introduction

Patients with advanced cancer are faced with many decisions during the course of their illness trajectory. There is much at stake for these patients as they weigh the toxicity of a particular course of treatment with potential benefits. These decisions are exceedingly complex and there many factors for patients to consider including their own goals of care, disease state, social support, treatment preferences, advance care planning, and others [1–3].

The benefits of shared decision-making between the patient and health care provider as well as end of life (EOL) care discussions with patients have been extensively reported in the literature [4–6]. However, family caregivers play an important role in not only providing care to the patient but also as a source to help guide the patient with these difficult treatment decisions. While research has been conducted on patient family caregiver decision making within the context of dying patients [7–8] and patient and family caregiver preferences for involvement in treatment decisions [9–10], to date, there have been no reports comparing patients’ preferences and family caregivers’ preferences for the patients’ goals of care while making treatment decisions in an outpatient setting.

## Purpose of the Study

To better understand the nature of the relationship between patients’ and caregivers’ preferences for goals of care, we aimed to examine the concordance of patients’ and caregivers’ goals of care for the patient at enrollment into the study and prior to death.

## Methods

Data were collected from a sub-sample of an on-going longitudinal descriptive study. The parent study (n=378), employs complexity theory to examine factors that influence cancer treatment decisions and aggressiveness of care at EOL.

All data were collected in the outpatient ambulatory clinics of a large, urban Midwestern National Comprehensive Cancer Center. A total of 237 adult patient and family caregiver dyads were enrolled in the study. Inclusion criteria included a diagnosis of advanced gastrointestinal or lung cancers and the patient had to have a designated family caregiver. All patients had a diagnosis of stage IV cancer with the exception of those with adenocarcinoma of the pancreas (Stages I-IV). Data were collected every three months for a total of 15 months or until the death of the patient. For this report, we utilized two data points for analysis—at enrollment in the study and the last interview prior to death.

Prior to data collection, written informed consent was obtained from the patient and family caregiver. The study was approved by the hospital Institutional Review board. At each time point for data collection the patient was asked, “Regarding your care, what is most important to you right now?” and indicated their response on a visual analog scale (VAS) where 0 represented comfort and quality of life preference and 100 represented survival and length of life preference. The caregiver was asked a similar question, “Regarding the care your loved one receives, what is most important right now?” and answered using the same VAS where higher numbers indicated a preference for length of life over quality of life. At enrollment, demographic and clinical data were collected using interview and chart review from the electronic medical records.

We used IBM SPSS Statistics 24 for all statistical analysis. Descriptive analyses (means, frequencies, percentages) were used to describe demographic and clinical data and comparisons were made using paired sample t-tests. A P value of < .05 was considered statistically significant.

## Results

### Sample Description

A description of our sample of 237 patient and family caregiver dyads is presented in Table 1. A majority of the patient sample was Caucasian with an annual household income >$50,000/year. Most patients had no prior history of cancer, a diagnosis of gastrointestinal cancer, had an ECOG status of 0 or 1 and were not employed. Caregivers were predominantly female, the spouse of the patient, and middle-aged. Almost half of the caregivers were employed with more than a quarter of those having had to reduce their work hours due to caregiving responsibilities.

### Concordance Between Patients and Family Caregivers’ Preferences for Goals of Care

**Figure 1:**
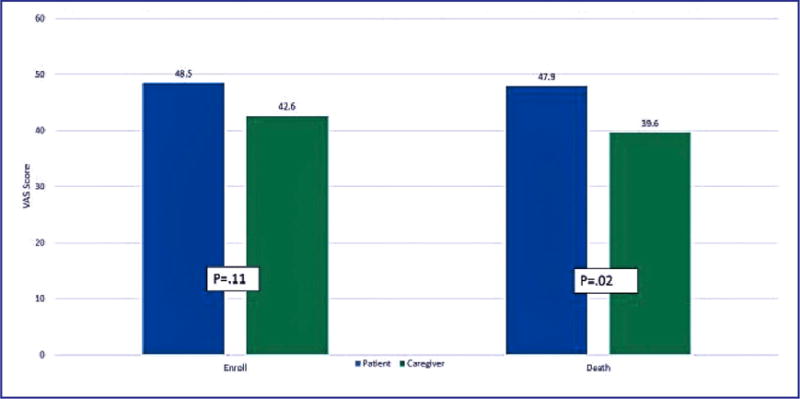
Preference Scores: Paired T-tests VAS: QOL Exclusively=0, Survival Exclusively=100

Preferences for goals of care in terms of quality of life versus length of life, were assessed at enrollment into the study and at the last interview prior to the death of the patient (Figure 1). At enrollment, patients were more likely to prefer a treatment plan focused on length of life than their family caregivers but the difference was not statistically significant (p=.11). Proximal to death, the difference in goals of care between patients and caregivers were statistically significant (p=.02) with caregivers favoring goals more closely aligned to comfort care (M=39.6) than the patients’ goals (M=47.9).

## Discussion

Our results showed that patients were more likely than their family caregivers to favor goals of care focused on length of life over comfort/quality of life at the EOL. As patients moved closer to death, the differences in goals of care between the patient and family caregiver became more pronounced. It is possible that this difference in goals of care at EOL could impact the aggressiveness of care that the patient received and may also contribute to caregiver distress or decision regret after patient death [11]. Further work needs to be done to examine the impact of this discordance upon caregivers and the care that patients receive at EOL. In addition, we must continue to find meaningful ways for family caregivers and patients to discuss goals of care throughout the cancer trajectory [12–14]. The communication skills that nurses bring to the interdisciplinary health care team put them in the ideal position to initiate and facilitate these discussions with patients and family caregivers [15–16].

## Limitations

Our study had several limitations including our limited cancer types and single study site at a tertiary academic medical center. Given the differences in various settings, these findings may not represent all patients and family caregivers. In addition, we used a single one-item VAS to assess goals of care. A more in-depth qualitative component could provide additional insight into sources of agreement and discord between patient and caregiver goals of care over time.

## Figures and Tables

**Table 1:** Sample Demographic and Clinical Characteristics (n=237)

Variable	Patient	Family Caregiver

Age: M (SD)/years	64.6 (10.6)	59.4 (12.3)

Self Rating of Physical Health Status: M (SD) (0–100: Higher scores=Better Health)	64.9 (22.1)	77.8 (20.0)

	**N (%)**	**N (%)**

Gender: Female	107 (45.1)	164 (69.2)

Race: Caucasian	196 (82.7)	198 (83.5)

Marital Status: Married	173 (73.0)	188 (79.3)

Relationship to Patient		
Spouse Child Sibling Other	------	152 (64.1) 39 (16.5) 18 (7.6) 28 (11.8)

Type of Cancer:		
GI	154 (65.0)	------
Lung	83 (35.0)	

Annual Household Income		
< $20,000	37 (17.3)	18 (8.5)
$21,000 – $49,000	66 (30.8)	50 (23.5)
> $50,000	111 (51.9	145 (68.1)

ECOG		
0	54 (22.9)	
1	144 (61.0)	------
2	27 (11.4)	
3	11 (4.7)	

Clinical Trial Participation: No	192 (81.7)	

History of Prior Cancer: No	198 (83.9%)	------

Employment Status: Employed	55 (23.3)	117 (49.4)

Reduced Work Status in Last 6 Months: Yes	------	32 (27.4)
